# The Examination of the Foeces as an Aid to Diagnosis

**Published:** 1908-08

**Authors:** Jos. N. Le Conte

**Affiliations:** Lecturer Diseases of the Stomach, Atlanta School of Medicine. Gastrologist to the Presbyterian Hospital, Atlanta, Ga.


### THE EXAMINATION OF THE FOECES AS AN AID TO DIAGNOSIS.*

DR. JOS. N. LE CONTE, LECTURER DISEASES OF THE STOMACH, ATLANTA SCHOOL OF MEDICINE. GASTROLOGIST TO THE PRESBYTERIAN HOSPITAL, ATLANTA, GA.

My excuse for opening this odorous subject is the great practical importance which it has attained in the past few years, and the scant attention it had previously received at the hands of the clinician.

Read before Fulton County Medical Society, June 4th 1908

As Schmidt very aptly expressed it, "Scarcely any practitioner has proceeded differently from the layman in the investigation of the foeces—that is, he inspects it, smells at it, and at most stirs it with a stick."

One reason for this neglect has been the lack of any systematic plan of examination which was sufficiently rapid, simple and reliable for the clinician to employ, and an interpretation of these findings based upon a large number of examinations.

It was while working in the laboratory of Adolf Schmidt in Dresden in the winter of 1905-1906 that I became familiar with his functional test of the intestines, which is now being accepted both in this country and Europe as the standard. This functional test was developed along the same plan as the test breakfast in gastric diagnosis, and only after much tedious experiment and modification was a test diet developed which conformed to the following requirements: (1) that it should be easily digested, both by a healthy person and one with intestinal disturbance, (2) should contain the requisite number of calories, (3) should contain carbohydrates, fats and proteid in the porper proportions, (4) the food stuffs should be easy for the patient to obtain and prepare, and lastly the method of examination should be simple, quick and in reach of the busy practitioner; and it is this standardized test diet and Schmidt's collated findings in the resulting foeces, both in health and disease, which has furnished a working basis for the investigation of the functions of the stomach and intestine.

The results are still far from ideal, and much remains to be done in bringing this work to the degree of accuracy now attained in testing the gastric functions, but we must remember that it is about ten years behind the corresponding work which has been done upon the stomach.

The daily diet as now recommended by Schmidt, and his coworker, Strassburger, consists of one and one-half quarts of sweet milk, three and a half ounces of zwieback or toasted stale white bread (avoirdupois weight is used here), two eggs, one and a quarter ounces of butter, four and a half ounces of chopped lean beef, six and a half ounces of mashed Irish potatoes and three ounces of oatmeal flakes. In order to meet the American plan of three meals daily it may be arranged as follows:

*Breakfast.*—One glass of milk, four pieces of zwieback or two slices of toast bread, one very soft boiled egg, butter size of walnut, a pint of strained oatmeal gruel (made from three ounces of oatmeal flakes, one glass of sweet milk and one glass of water).

*Dinner.*—Two glasses of sweet milk, four pieces zwieback, or two pieces dry toasted bread, butter, four and a half ounces of chopped beef lightly broiled in butter so that the center is still red and rare, six and a half ounces of mashed potato.

*Supper.*—Two glasses of milk, four pieces of zwieback or two pieces toasted bread, butter, one very soft boiled egg.

If milk disagrees, or is repugnant to the patient it may be reduced by one-half and the remainder served at breakfast and supper as milk-cocoa and cooked with the oatmeal gruel. Patients often complain that the diet is too bountiful, and unless quantitative tests of the excrement are being made it is not necessary for the patient to take the entire quantity of the diet, but it *is* important for the accuracy of the test that the full quantity of the carbohydrates be taken and that the center of the meat ball shall be rare.

This diet should be continued for three or four days, a stool on the third day of the diet being generally suitable for examination. If desired, 5 grains of carmine may be given at the beginning and end of the diet which will indicate by its red color the test excrement. The patient is directed to send the entire stool in a fruit jar or other closed receptacle as soon as possible after evacuation.

The macroscopic examination often reveals points of importance. The shape, size, color and consistency should be noted, and especially shreds or masses of mucous filling the interstices of the formed stool. Only the large, formed, sausage shaped is normal; other forms are generally pathologic. The stool should be thoroughly mixed with a wooden paddle and a portion of the size of a walnut placed in a mortar and very thoroughly ground up with the gradual addition of distilled water until it is of a fluid consistency, and then spread on a black and white plate—one can use to advantage a porcelain or white enamel plate, one-half of which has been painted black. Normally we see only a few brown points scattered over the surface, whereas we may find pathologically: (1) Glassy or membranous pieces of mucus of varying size, the smaller particles lying flat on the plate. (2) Connective tissue threads, white, elastic, and which cannot be teazed apart with a needle. (3) Small brown masses or rolls of muscular tissues, cheesy and friable, showing striation under the microscope. (4) Residue of potato in glassy grains resembling particles of mucus, but they stand above the level of the layer and under the microscope show empty potato cells and particles of starch, staining blue with iodine.

The microscopic examination is of value criefly to verify and strengthen the macroscopic findings.

Three specimens from the mixed foeces are placed on a slide, one is examined plain, after being crushed to a thin layer with the cover glass; the second specimen is mixed with one drop of 30 per-cent. acetic acid and heated until it begins to boil, then covered with a cover glass; the third is mixed with a drop of iodine solution prepared by mixing iodine one, potassium iodid two and distilled water fifty parts.

The first mount will normally show fine residue and at times isolated muscle fibres, yellow with round edges and showing no striation or very faint; also yellow fatty acid salts of calcium, colorless soaps in shell-like shapes, occasional empty potato cells and hulls from the oatmeal grains.

The acetic acid mount brings out all of the fat contents, and normally we see a moderate amount of small fatty acid crystals scattered over the field.

In the iodine mount we normally see a brown field in which may apepar a few remains of starch stained violet from the iodine.

Pathologically we may find: (1) In the plain preparation an increased quantity of muscle in bundles with sharp edges and distinct striations, islands of neutral fat which glow with refracted light when the mirror is shaded by the hand, large numbers of small fatty acid and soap needles, many potato cells containing starch, parasite eggs, mucus, connective tissue. Charcot Leyden crystals if seen, speak for the presence of worms, but are not pathognomonic. (2) In the acetic acid mount the field may be dominated by large fatty acid crystals. (3) In the iodine mount excessive quantities of blue starch, various fungus spores and bacilli strained blue, and yeast cells, which are colored yellow by iodine.

For routine work the chemical examination consists only of reaction, the fermentation test and the test for unchanged bile pigment.

Red and blue litmus paper is floated on the ground-up fluid stool, and normally should be amphoteric, faintly acid, or faintly alkaline.

For the bile pigment test some of the ground up foeces is thoroughly mixed in a small Petri dish with a considerable amount of watery solution of corrosive sublimate (Corros. Sublimate 50, Sod. Chloride 5, Dist. Water 1,000), and allowed to stand for twenty-four hours at room temperature. We normally get a red color, due to the hydro-bili-rubin, but a green color, or even very small green particles seen miscroscopically, indicate unchanged bilirubin, and is pathological.

For the fermentation test an instrument is used similar in principle to Einhorn's saccharometer, in which the fermentative gases displace the water in an inverted test tube and drive it into an open tube placed parallel and connecting with the first.

A portion of the undiluted mixed foeces of the size of an olive is placed in the lower vessel and mixed with water, the rubber stopper is pressed down until all air is excluded, the inverted closed tube is filled wtih water and fitted on and the open ascension pipe is left empty. The apparatus is then placed in a thermostat at 98 degrees Far. for 24 hours; at the end of this time the height of water in the open ascension pipe is read off. If more than one-half of the tube is occupied by water it is deemed pathological. Schmidt first placed the pathological limit at one-fourth, and in his last book at one-third, but he has since told me that he only considers one-half or more as pathological.

The litmus reaction should be taken before and after fermentation. In carbo-hydrate fermentation the diluted foeces has become more acid, is lighter in color and the gas in the tube has the odor of butyric acid. In putrefactive processes there is relatively less gas, which is foul in odor, the foeces are darker in color and more alkaline in reaction than before fermentation.

Clinically one of the most important pathological findings in the stool is mucus, which when present always indicates an inflammatory process of the intestines, though inflammation may be present without mucus being demonstrable. The mucus visible in the foeces is almost invariably from the colon, and the smaller the particles and the more thoroughly mixed with the foeces, the higher its origin. I have often seen abundant mucus revealed in the black plate after grinding up, when it had not been visible on inspection. In these cases the mucus was probably freshly formed high up in the colon, and became incorporated in the foeces while the latter was still in a liquid state.

Mucus which is formed in the small intestine is generally digested before it reaches the rectum, and may only be assumed to be present in the foeces in cases of, (1) liquid foeces, with, (2) rapid peristalsis, where, (3) very small mucus particles show microscopically many bacteria and half digested cells, (4) if the sublimate test is positive, and if, (5) much muscle and potato is visible to the naked eye.

In the sublimate test a green color of all of the foeces, or even miscroscopic points generally indicates too rapid peristalsis with probable involvement of the small intestine. Complete absence of either red or green color indicates complete absence of bile, from obstruction or suppression.

The presence of connective tissue in any quantity indicates disturbance of stomach digestion, generally a deficiency or absence of HCL, and Pepsin, though in rare cases an excess of HCL, seems to inhabit the development of Pepsis and prevent the gastric digestion of raw connective tissue. Schmidt has found that in the digestive tract, only the stomach is capable of digesting raw connective tissue, and on this account the test diet may be used to advantage in those cases where it is impossible to introduce a stomach tube.

In a large number of cases where I have examined the test diet stool, and also examined the stomach contents, the presence of connective tissue in the ground-up, diluted foeces was always acompanied by gastric hypochylia or achlyia, the foeces apearing when stirred as if fine cotton fibres were incorporated in it. If muscle fibres be visible, or an excessive amount be seen miscroscopically it indicates some disturbance of the small intestine or pancreas.

Pronounced albumin putrefaction in the fermentation test, as shown by small amount of gas formation, foul odor to the gas and increased alkaline reaction speaks for a severe digestive disturbance which is connected with organic disease of the intestinal mucus membrane, the inflammatory products of which (mucus, serum, pus) are the promoting cause of the putrefaction. Experiments have shown that undigested albumin food remains in a digestive tract which has undergone no pathological changes, do not develop high degrees of putrefaction. This would go far to explain the high degree of putrefaction seen in intestinal catarrhs, as shown by the fermentation tube, excessive indican in the urine and the general toxemia. A good illustration of the important relation which foecal investigation bears to all branches of medicine, is the pioneer work done by Drs. Hoke and Andrews of this city showing the causal relation of intestinal putrefaction to toxic arthitis, and their experience proves that great benefit may accrue to the patient by combatting the intestinal disturbance, in some cases directing no treatment whatever to the joints,

Fermentation Test.—Unfortunately a negative result does not assure us positively that all starch is being digested; only the positive result is of value.

Einhorn has devised an ingenious test of the general digestive capacity, by means of various food stuffs tied to beads and allowed to pass through the digestive tract. Particles of raw catgut, fish bone, raw beef fiber, raw thymus gland, mutton fat and partly cooked potato are fastened in pairs to three glass beads, which in turn are tied on a string; the whole is put in a gelatin capsule and administered to the patient. The string of beads is recovered by means of the stool sieve, and examined as to what materials have been digested. This test will probably be of use in selected cases, but does not furnish the accurate test of digestion afforded by Schmidt's test diet, which in kind, quantity and caloric value, measures up to an average normal food diet.

If the test stool shows excess of fats, visible muscle tissue and normal bile pigment we may suspect pancheatic disturbance, though it often exists with no abnormality of the foeces.

Many attempts have been made to devise some reliable method of determining the kind and severity of pancreatic disturbances, but none of these have succeeded in revealing the slight functional troubles. Schmidt found by experiment that nuclii of tissue cells were only digested by the pancreatic juice, and upon this based his test of pancreatic function. Small cubes of fresh raw beef about 1-8 of an inch square are hardened in alcohol, placed in small bags of gauze and given to the patient with the noon meal of the test diet. The bags are then removed from the foeces with a stool sieve and the meat remains stained and examined microscopically for cell nuclii. If all or the greater part of the nuclii are preserved we have a very severe disturbance of pancreatic secretion. More than this we cannot say, as the nuclei may be destroyed by putrefaction in cases of constipation, and moreover it has been found that mild disturbances of the pancreas may be present and still the nuclei be digested.

Sahli's glutoid capsule test, which was devised for the same purpose, consists of a gelatin capsule containing 2gr. of iodoform, the capsule being treated with Formaldehyde to render it insoluble in the gastric juice, but not insoluble in the normal pancreatic juice, the capsule being administered with an Ewald test breakfast, it passes from the stomach to the intestine, is dissolved by the pancreatic juice, and iodine in the saliva may normally be revealed in from fifteen to seventy-five minutes. Certain sources of error have been found in this test, however, so that only the postitive test is of value. A prompt and normal iodine reaction in the saliva positively indicates normal pancreatic secretion.

This test combined with the nuclei test informs us as to the extremes of normal and purverted function, but the ground between these two is as yet unexplored.

The examination of the foeces for parasites or their eggs, such as the Taenia, Ascaris, Oxyuris, has long been in general use, and the work of Stiles in the study of Uncinariasis has been of the greatest value, especially in this section of the country where the disease is so prevalent. Probably no research work of recent years has been of more social and economic value than this, which often explains and remedies the anaemia and arrested development of a whole settlement of cotton mill operatives or school children.

In those cases where parasites or their ova, or microorganisms, such as the amoeba coli are to be sought for, solid or liquid foeces may be conveniently obtained for microscopical or other examination by the introduction of Cohnheim's foeces extractor, a small glass cylinder closed and bulbous at one end and having a smooth eye for the collection of the foeces, which may be examined while still fresh and warm.

Where the larger parasites, gall stones, entero-liths or foreign bodies are to be sought for, a stool sieve should be employed, the best type of which is Strauss' instrument, consisting of a glass jar like an inverted bell glass covered at the top with a sieve, the foeces being placed in the jar, tap water is carried by a rubber tube to the bottom of the jar, beats the foeces to pieces, the water overflowing the jar carries with it the finer particles and in this way prevents the obstruction of the sieve, which is a defect of other types.

The tests for occult blood in the foeces are often of value in diagnosis, though we must decide from other symptoms from what point of the gastro-intestinal tract the hemorrhage arises. Observation has shown that in a large percentage of cases of gastric carcinoma occult blood will be regularly found in the foeces day after day, whereas in gastric ulcer it will be present one day and absent the next, therefore requiring repeated examinations in order that a positive or negative result may be of value. These tests for blood are often positive in a healthy person after the ingestion of rare of even well cooked meats, and therefore all flesh should be excluded from the diet for three or four days before the test is made.

The guaic or aloin tests are the most useful, the benzidin test being more sensitive, but reacting also to many other materials besides blood.

VanDeen's modification of Weber's Guaiac test is performed as follows:

A portion of the mixed foeces the size of an olive is rubbed to a mushy consistency with distilled water, to which is added one-third of its volume of glacial acetic acid and shaken, an equal volume of sulphuric ether is added and carefully shaken through. The ether is then decanted. One part of resin of Guaiac or chips of guaiac wood are heated with twenty-five parts of absolute alcohol, and ten drops of this guaiac tincture added to 2cc of the ether extract, and drop by drop, 20 to 30 drops of old ozonized oil of turpentine. If haematin is present a blue color of varying intensity appears, which fades in a few minutes.

A point which is often overlooked is that the guaiac extract shall be freshly made and the turpentine must be old and ozonized, or the test is unreliable.

As a substitute for the guaiac one may use a knife point (5gr) of aloin dissolved in 10cc of 70 per cent alcohol, and proceed as above, the positive reaction showing a deep cherry red.

I have employed the Schmidt function test of the intestines in a large number of cases in the past two years, and I am convinced that its value as a diagnistic aid ranks equally with the analysis of the gastric contents in diseases of digestion and metabolism, indeed one test is the complement of the other in the investigation of these conditions.

References:

Schmidt und Strassburger, Foeces des Menschen, 1905.

Schmidt. Funktions prufung des Darmes mittels der Probekost, 1904.

Sahli. Lehrbuch der klinischen Untersuchungs Methoden, 1905.

Strassburger. Die Untersuchung der Foeces. Deutsche Klinik, 1905.

Einhorn. Digestive Beadtest, etc., Jour A. M. A. February 2, 1907.

